# The Scientist Network for Advancing Policy (SNAP): building infrastructure for early-career scientists in policy and public engagement

**DOI:** 10.1093/biosci/biag053

**Published:** 2026-06-18

**Authors:** Miles Arnett, Dimitris Boufidis, Cael Dant, Brendon Davis, Isako Di Tomassi, Kassandra Fernandez, Amanda Finn, J P Flores, Saul J Flores, Alex Lando, Erin Morrow, Disha Patel, Anna Rader Groves, David Ramotowski, Alex Rich, Emma Scales, Lani Tran

**Affiliations:** Scientist Network for Advancing Policy; Department of Bioengineering, University of Pennsylvania, S 33rd St., Philadelphia, PA 19104, USA; Scientist Network for Advancing Policy; Department of Bioengineering, University of Pennsylvania, S 33rd St., Philadelphia, PA 19104, USA; Scientist Network for Advancing Policy; Program in Plant Biology and Conservation, Northwestern University, 2145 Sheridan Road, Evanston, IL 60208, USA; Scientist Network for Advancing Policy; Department of Biology, Johns Hopkins University, 3400 N Charles St., Baltimore, MD 21218, USA; Scientist Network for Advancing Policy; School of Integrative Plant Science, Cornell University, 236 Tower Road, Ithaca, NY 14853, USA; Scientist Network for Advancing Policy; Department of Engineering Education, University of Florida, 1929 Stadium Road, Gainesville, FL 32608, USA; Scientist Network for Advancing Policy; Department of Nutrition Sciences, University of Alabama at Birmingham, 1675 University Blvd., Birmingham, AL 35294, USA; Scientist Network for Advancing Policy; Bioinformatics & Computational Biology, University of North Carolina at Chapel Hill, 120 Mason Farm Road, Chapel Hill, NC 27514, USA; Scientist Network for Advancing Policy; Department of Chemistry and Biochemistry, University of Maryland, 8051 Regents Dr., College Park, MD 20742, USA; Scientist Network for Advancing Policy; School of Integrative Plant Science, Cornell University, 236 Tower Road, Ithaca, NY 14853, USA; Scientist Network for Advancing Policy; Department of Psychology, University of California Los Angeles, 740 Westwood Plaza, Los Angeles, CA 90095, USA; Scientist Network for Advancing Policy; Department of Chemistry and Biochemistry, University of Notre Dame, 251 Nieuwland Science Hall, Notre Dame, IN 46556, USA; Scientist Network for Advancing Policy; Neuroscience Institute, Georgia State University, 100 Piedmont Ave. SE, Atlanta, GA 30303, USA; Scientist Network for Advancing Policy; Civil and Environmental Engineering, University of Iowa, 4105 Seamans Center for the Engineering Arts and Sciences, Iowa City, IA, IA 52242, USA; Scientist Network for Advancing Policy; Department of Neuroscience, Yale University, 100 College St., New Haven, CT 06510, USA; Scientist Network for Advancing Policy; School of Integrative Plant Science, Cornell University, 236 Tower Road, Ithaca, NY 14853, USA; Scientist Network for Advancing Policy; Neuroscience Graduate Group, University of Pennsylvania, 3620 Hamilton Walk, Philadelphia, PA 19104, USA

**Keywords:** science policy, civic engagement, graduate STEM education, early-career scientists, evidence-informed policymaking

## Abstract

The Scientist Network for Advancing Policy (SNAP) is a trainee-led grassroots coalition founded to empower early-career researchers to engage meaningfully with science policy, advocacy, and public-facing science communication. Our mission is to equip scientists with the tools, networks, and confidence to translate their research and expertise in service of their communities. In this Special Report, we share how SNAP’s initiatives, spanning from local community conversations to national policy debates, are reimagining and transforming the multifaceted role of scientists in communities. By investing in coalition-building and centering early-career voices, SNAP is creating scalable models for civic engagement and science advocacy to advance equity, democratize science policy, and strengthen the relationship between science and society.

## The need for the Scientist Network for Advancing Policy

Scientists across disciplines increasingly recognize that their work sits at the intersection of discovery and societal decision-making (Bednarek et al. 2018, Gluckman et al. 2021). Yet, most academic training leaves scientists underprepared to navigate the complex terrains of policy, governance, and civic discourse (Bushana et al. 2019, National Academies of Sciences, Engineering, and Medicine 2018, Tyson and Kennedy 2024). The Scientist Network for Advancing Policy (SNAP) seeks to embed civic and community engagement into the culture of scientific training and practice (Singh et al. 2019). Our coalition was created in 2025 by early-career researchers who recognized the urgent need for infrastructure to support scientists in developing policy literacy, advocacy, and communication skills, as well as networks of mutual support among scientists and local communities (Balazs and Morello-Frosch 2013, Pandya et al. 2025).

## Ethos and approach

At its core, SNAP operates through coalition-building: we are not a professional society with top-down governance, but rather a networked infrastructure built by and for early-career scientists. SNAP is nonpartisan and aims to strengthen evidence-informed civic engagement across communities and political contexts. Our work is guided by values of equity, reciprocity, and collective action and impact. Like recent efforts to shift scientific societies toward braided-river models of inclusion (Batchelor et al. 2021, Alemán-Díaz et al. 2025), SNAP embraces nonlinear pathways into science policy and values diverse forms of expertise.

## Early work: the foundational initiatives of SNAP

### The McClintock Letters

The McClintock Letters initiative was SNAP’s first major effort aimed at humanizing scientists and democratizing science communication (Cornell Advancing Science and Policy Club, n.d., Drake 2025a, Fortin 2025, The New York Academy of Sciences 2025). The initiative was born from a simple yet powerful idea: we can address misunderstandings about federally funded research by encouraging scientists to speak directly to their own communities about how they use federal funding (Klein and Palma Kimmerling 2025).

The McClintock Letters initiative draws inspiration from Dr. Barbara McClintock, the first American woman to win a solo Nobel Prize in the sciences (Nobel Prize Outreach 2026). Her legacy as an early advocate for foundational science funding inspired the early SNAP team to act amid proposed cuts to federal science funding, the restriction of fundable research subjects, and the widespread firings of federal scientists in early 2025 (Tollefson 2025, U.S. Senate Permanent Subcommittee on Investigations, Minority Staff 2025, Kozlov et al. 2026).

**Figure 1.**
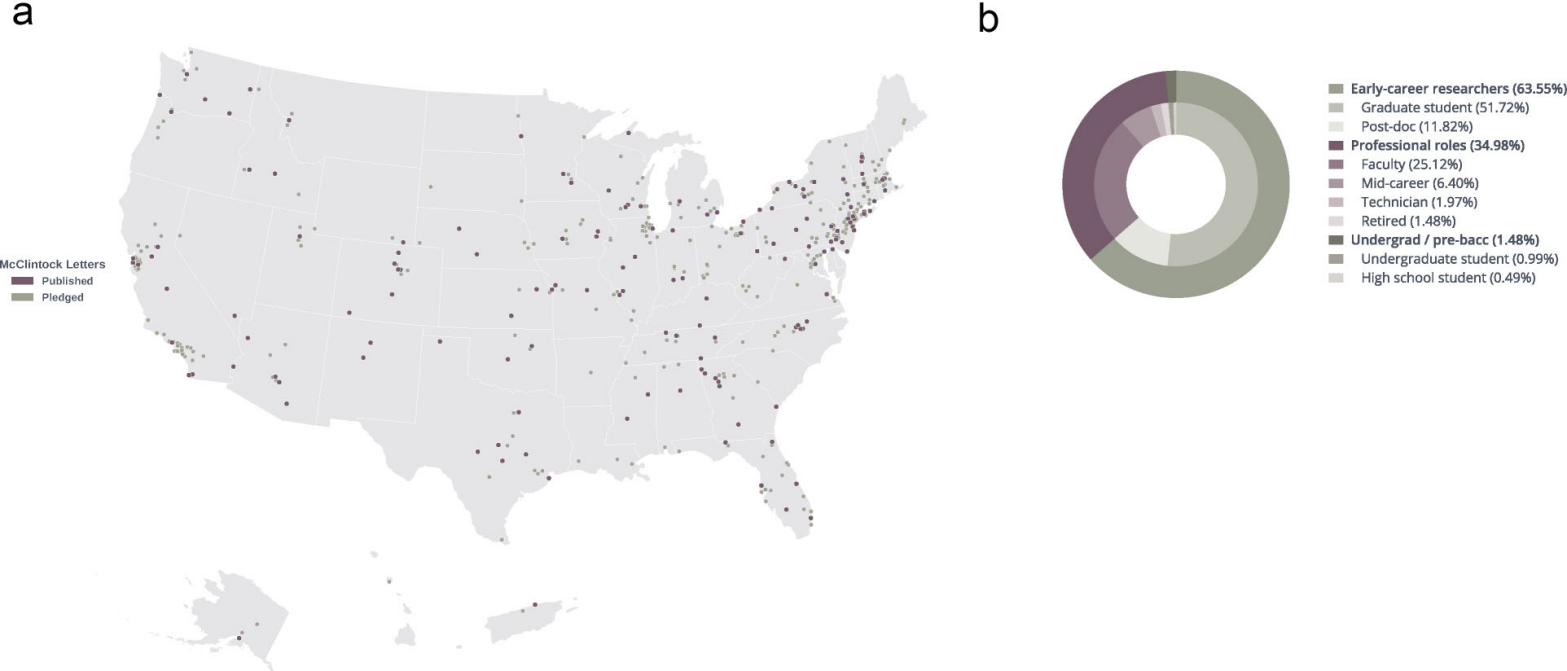
McClintock Letters. (a) Confirmed locations of newspapers in which writers pledged to and/or did publish McClintock Letters. “Pledged” indicates a commitment from participants to write, whereas “published” indicates a confirmed publication in a local newspaper. Note: Many of the “pledged” writers may have participated in training, published their Letters without sharing them with SNAP, and/or published through other outlets, including *SciTech Forefront*, which hosts letters that were not published elsewhere (SciTech Forefront). (b) Distribution of writers by career stage.

Researchers wrote op-eds for their hometown newspapers, reflecting not only on their work but also on the places and communities that shaped their journey into science. Participants were encouraged to share their work’s real-world impacts and to close with a call for civic engagement—for example, urging readers to contact elected officials in support of research funding. So far, over 200 McClintock Letters have been published in local newspapers in at least 45 states, Washington, DC, and Puerto Rico (figure 1a). Although the reach of this initiative grew to include significant participation from university faculty members, most participants who published letters were early-career researchers (figure 1b).

To achieve results of this scale as a decentralized, grassroots collective, we leveraged our network to accomplish the following goals:

1. **Generate ideas**. The McClintock Letters initiative was conceptualized by SNAP members who lead the Cornell Advancing Science and Policy (ASAP) Club, inspired by the mission behind Science Homecoming, to help scientists write opinion pieces advocating for the importance of science in their hometown newspapers (Science Homecoming 2025). We differentiated the McClintock Letters by focusing on early-career trainee perspectives and community connections, providing virtual writing workshops, and organizing a mass effort to publish pieces around June 16th, Dr. Barbara McClintock’s birthday (Nobel Prize Outreach 2026). The initiative represented what we felt would be most impactful in the wake of our personal experiences following unprecedented federal cuts to science in early 2025 (Wadman and Kaiser 2025).
2. **Maximize impact by leveraging collective effort**. The newly formed nationwide SNAP coalition scaled ASAP’s initial concept to facilitate national reach. This collaboration with other early-career researchers across the United States provided new ideas and refinement, a unique knowledge base, and manpower.
3. **Garner participation**. We sent hundreds of cold emails with recruitment flyers (Supplemental figure 1) to research institution administrators, department heads, graduate student groups and unions, professional scientific societies, and other hotbeds of researchers with the request to distribute our materials to their networks. As a result, thousands of scientists were exposed to the initiative; over 600 pledged to participate, more than 200 of whom shared their published letters with us (figure 1a). We built a network of motivated scientists, many of whom participated in later SNAP initiatives.
4. **Fill training gaps to ensure success**. We utilized the expertise of SNAP members and their connections to organize several virtual and in-person trainings that covered op-ed writing and skills for communicating science to a nonexpert audience. These trainings served an estimated 300+ participants. We also offered a dedicated Slack space for writers to share ideas and get feedback on their op-eds from peers.

Collectively, the accumulation of hundreds of voices across the nation helped weave a tapestry of the sweeping collective influence of scientists. For SNAP, the McClintock Letters served as both a narrative tool *and* an organizing lever—transforming individual stories into threads of public trust and civic connection with McClintock Letter readers and writers.

Ultimately, the far-reaching outcomes of the McClintock Letters exemplify the power of grassroots efforts grounded in sincerity and altruism. The overwhelming positive response from participants shows that early-career researchers are eager to gain skills beyond the lab bench and engage in efforts to bring their science to the public. Public response was also overwhelmingly positive, with over 200 responses to the McClintock Letter reader pledge that showed readers’ interest in reaching out to public officials. We hope the McClintock Letters initiative serves as inspiration for the integration of meaningful public-facing science communication as part of the standard operation of research institutions and scientist training.

### Congressional District visit days

Building on the success of the McClintock Letters initiative, SNAP created another opportunity for science advocacy during the congressional August recess (Senate Historical Office 2023). Many SNAP members had taken part in DC-based science advocacy events through the support of scientific societies in the past. By relocating advocacy opportunities from Washington, DC, to district offices, however, SNAP’s Congressional District Visits initiative lowered barriers to participation, built community connections, and emphasized that science advocacy could be done directly and locally in every congressional district (Drake 2025b).

To accomplish our goal of coordinating local congressional visits for early-career researchers in August, we followed a similar process to the McClintock Letters initiative, starting with preparation one month prior:

1. **Generate ideas**. Inspired by the strategies behind the annual American Institute of Biological Sciences (AIBS) Biological Sciences Congressional District Visits (AIBS 2025), SNAP imagined a congressional visits initiative open to early-career researchers from any discipline. With a congressional recess and deliberations of science agency appropriations on the horizon, SNAP set a goal to hold these meetings in late August to advocate for (1) sustaining or increasing federal funding to support scientific discovery, education, and innovation and (2) exercising congressional powers to halt destabilizing activities that threaten the continuity of research and the well-being of scientists.
2. **Maximize impact by leveraging collective effort**. The preparation for this initiative began with collaborations with the American Association for the Advancement of Science (AAAS) and AIBS. Members from these organizations gave feedback on the initiative and shared resources for participant training and contacting congressional staff. To create a manageable and regionally segmented approach to initiative coordination, SNAP members volunteered as leaders for each state, coordinating meetings and serving as a resource to participants.
3. **Garner participation**. We initially relied on our participant list from the McClintock Letters initiative to connect with policy-minded early-career researchers. To increase participation nationwide, we also sent invitations to student organizations and university and scientific societies.
4. **Fill training gaps to ensure success**. SNAP co-led a Congressional Visits 101 training session on Zoom with AAAS. Attendance at a training session was mandatory to ensure a unified message across all meetings. A total of 133 participants ultimately received training and joined a visit group. By mid-August, SNAP leaders for each state were scheduling congressional visits on behalf of the participant groups and preparing the group members for their visits.

**Figure 2.**
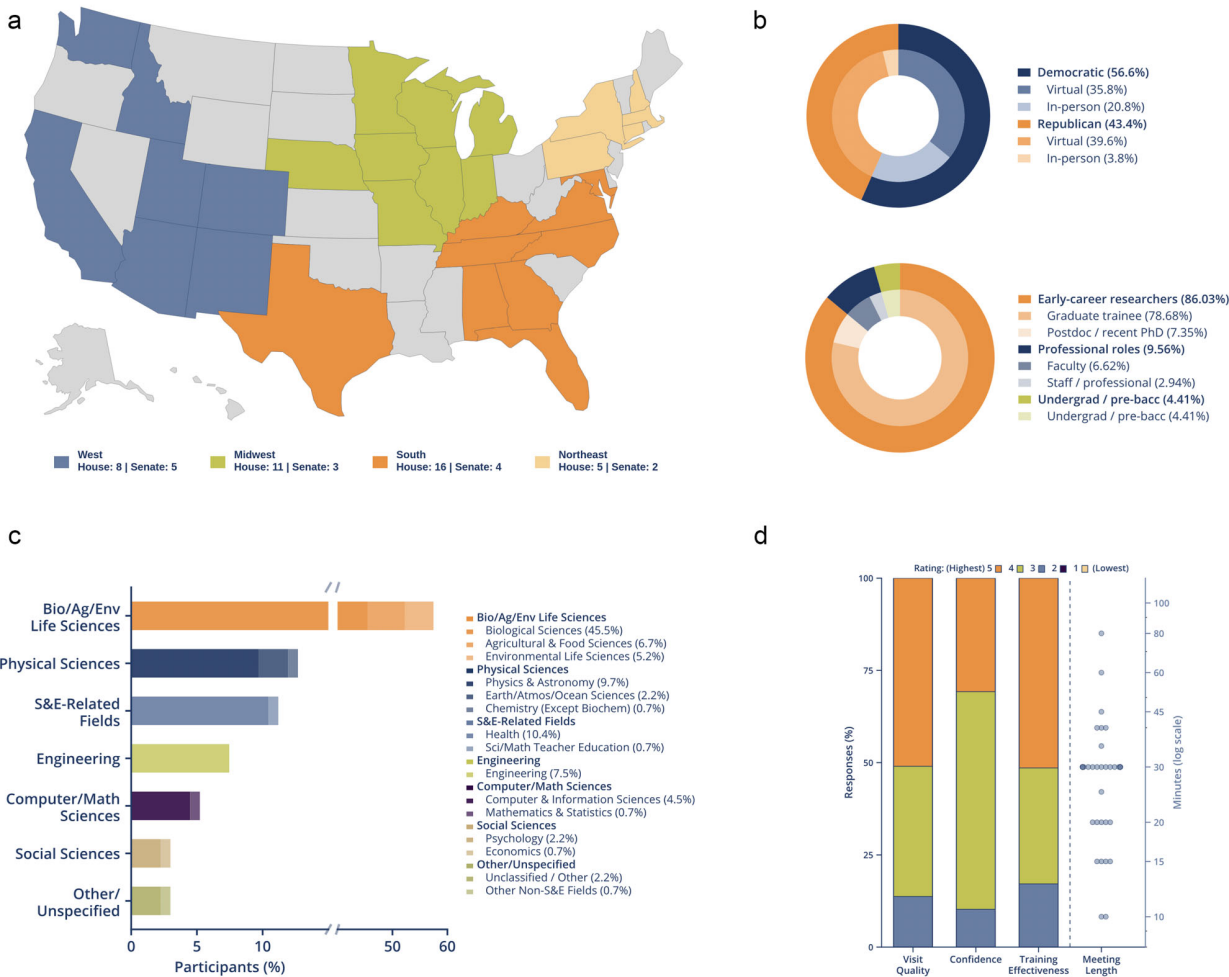
Congressional district days. (a) States in which meetings were held, color-coded by region. The figure legend details the distribution of meetings by chamber. (b) Distribution of political party and meeting format (top) and of participant career stage (bottom). (c) Areas of research expertise represented by participants. (d) Post-visit evaluations of visit quality, retrospective pre-meeting confidence, and perceived effectiveness of the training provided to participants. Meeting length is also shown, in minutes (right).

With the help of participants—and in some cases, highlighting quotes from their McClintock Letters—SNAP members created one-pagers tailored to each district for every scheduled meeting to ensure local resonance (Supplemental figure 2). These one-pagers highlighted testimonials and data underscoring how federal science dollars support workforce development, technology innovation, and community prosperity in the state and/or district. Participation in the initiative spanned 29 states, with meetings held in both US House and Senate offices (figure 2a). The 54 visits engaged offices across both Democrat- and Republican-represented districts and were conducted through a mix of virtual and in-person formats, with virtual meetings comprising the majority of visits to reduce barriers to participation (figure 2b). Participants represented a wide range of scientific disciplines and career stages, primarily graduate trainees and postdoctoral researchers (figure 2c). Post-visit evaluations indicated high satisfaction with visit quality, increased confidence in advocacy skills, and strong perceived training effectiveness, with meetings typically lasting 20–30 minutes, demonstrating the feasibility and value of in-district and virtual advocacy for early-career researchers (figure 2d).

Beyond the quantitative metrics, the impact of district visits lies in their civic imagination: They invite early-career researchers—graduate students, postdocs, and new faculty—to see themselves not only as scientists but also as constituents and community representatives, thus reinforcing the fact that science and community are irrevocably linked (Levine and McKnight 2025). Moreover, this initiative sought to increase equitable participation by reducing financial and other barriers to accessing policymakers through in-district and virtual visits. By directly engaging with lawmakers and building relationships at their district offices, this initiative helped reinforce scientific advancement as a shared, bipartisan value tied to local economies and everyday lives.

## Looking forward: building a civic-minded infrastructure for science

**Figure 3.**
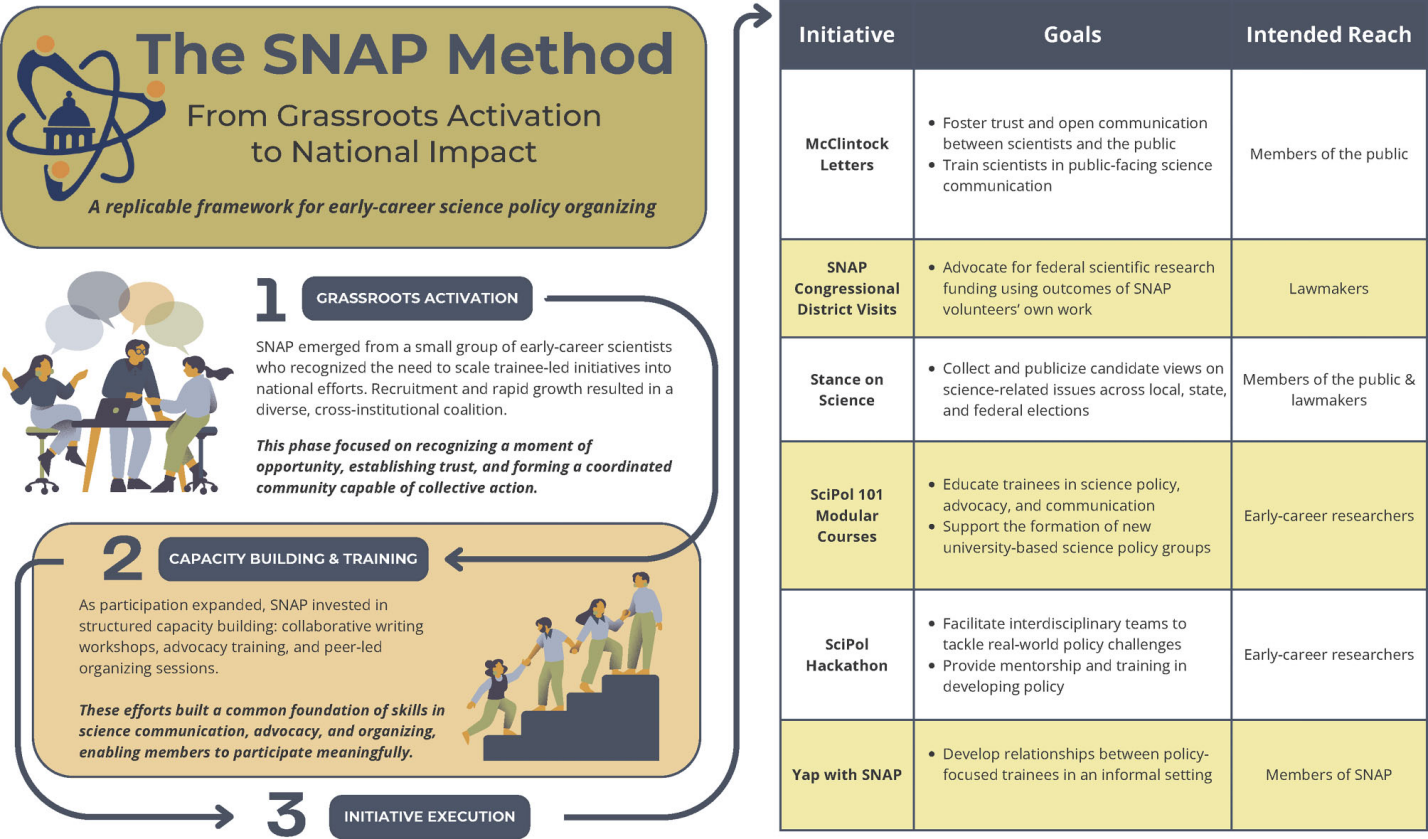
SNAP method. (Left) The basic framework for SNAP’s approach to organizing and implementing science policy initiatives. This method applies to the broader organization and to the execution of individual initiatives. (Right) Examples of previous and current SNAP initiatives. This chart illustrates SNAP’s multipronged approach to strengthening the discourse between scientists, policymakers, and the public.

Through grassroots activation and capacity-building efforts, SNAP is creating scalable models for community and civic engagement among early-career researchers to strengthen the relationship between science and society (figure 3). SNAP’s upcoming programs are organized around four complementary categories: public-facing science communication, science advocacy, early-career researcher training, and development of sustainable networks. Together, these initiatives ensure the SNAP method will be channeled into concrete outcomes for the future.

### Public-facing science communication

To engage lawmakers and the public, SNAP is expanding its public-facing science communication efforts. Through one of these new efforts, Stance on Science, SNAP has recruited volunteer-led teams to ask political candidates across local, state, and federal elections about their positions on science policy issues, particularly those related to early-career researcher support and scientific workforce development, through communication with candidates’ campaigns and town halls. Responses will be assessed and scaled using a nonpartisan rubric evaluating alignment with and openness to expert-informed, evidence-based policymaking. These assessments will then be widely publicized, with the intention of reaching the broader public and allowing voters to evaluate candidates’ science policy positions.

Complementing this work, SNAP has launched a blogging initiative, Science Policy in an SNAP, that provides a collaborative, grassroots platform for writing about science policy-related issues, current events, and SNAP initiatives while showcasing SNAP members and their work (Scientist Network for Advancing Policy n.d.). This is an ongoing initiative in the grassroots spirit of SNAP, allowing members to gain experience writing and editing articles that will be freely accessible to the public. The public can also comment on blog posts, building opportunities for dialogue between scientists and other community members.

### Science advocacy

Advocating for evidence-informed, science-backed policy is central to SNAP’s ethos. To this end, SNAP will further channel scientific expertise toward policy through its annual Science Policy Hackathon, launching in summer 2026. Inspired by coding hackathons, this event will bring together graduate students, postdoctoral researchers, and other early-career scientists to rapidly ideate and prototype policy solutions to pressing issues at the intersection of science and society. The first Hackathon will focus on policies that support and sustain the scientific workforce, such as graduate worker conditions, pathways into science policy careers, and mechanisms to broaden participation. SNAP will showcase the most promising ideas on public platforms. By channeling the creativity of early-career scientists into structured, time-bound policy innovation, the Science Policy Hackathon demonstrates SNAP’s ethos: policy engagement is not only possible but also generative, collaborative, and fun.

### Early-career researcher training

A persistent gap in scientific training is the absence of structured, accessible education in science communication and policy (Bushana et al. 2019, Singh et al. 2019, Schafer 2024). SNAP is helping to address this gap by developing a modular, open-access Science Policy 101 curriculum that can be adapted by universities, professional societies, or grassroots initiatives with attribution (Supplemental table 1). The curriculum is designed to educate trainees in the essentials of science policy and to encourage the formation of new university-based science policy groups.

SNAP’s open-access curriculum is a critical step toward realizing decades of discourse on how science communication and policy training can be formally incorporated into a scientific education. As PhD students ourselves, we recognize the difficulty in requiring additional training given the already intense demands of a graduate program. However, we also experience this as a meaningful gap in our development as scientists: the significance of the expertise we are building—and therefore, our responsibility—goes beyond knowledge creation to include how the knowledge we create is used and applied. To balance this, we argue that science communication and policy training should be embedded across all levels of scientific training, both before and beyond graduate school. By disseminating this open-access curriculum, we hope to enable a larger systemic change in which scientific education equips researchers to connect with the broader impacts of their work and the people who shape and experience those impacts.

### Sustaining networks

SNAP aims to sustain networks across initiatives using informal efforts such as Yap with SNAP, which provides regular sessions for members to chat outside of formalized agendas. This community-building creates accessible virtual spaces for early-career scientists to connect, share experiences, and build confidence. By encouraging continued participation across communication, advocacy, and training efforts, SNAP transforms isolated experiences into a connected, cross-institutional community.

## Conclusions

Members of SNAP are predominantly early-career researchers pursuing their scientific careers while creating outreach and advocacy opportunities to connect themselves and their peers with the public and lawmakers. The organization of these initiatives provides a strong foundation of professional and skills development, creating the space for SNAP members to continually learn and grow with one another. The organizers balance their research and teaching responsibilities with their advocacy and outreach efforts in the understanding that science discourse and translation are a two-way street between scientists and nonscientists.

SNAP represents a recent cultural shift in how scientists understand their roles in society. Our model illustrates that civic engagement is not an “add-on” but a core part of scientific practice, particularly for early-career researchers (Leshner 2003, Bushana et al. 2019, Singh et al. 2019, Gluckman et al. 2021). SNAP’s initiatives demonstrate that when scientists are equipped to engage other communities, they can strengthen democratic institutions, inform policy, and help ensure that science serves the public good.

A durable future for community-minded science depends on civic infrastructure that provides researchers with the skills, incentives, and institutional support necessary for meaningful engagement. Embedding civic engagement into scientific training can help create a generation of researchers who are not only knowledge producers but also trusted community partners and policy shapers. Some scientific societies, such as AIBS, offer awards and programming for science policy training to a handful of students every year. These programs generate a foundation of science policy leadership, but until they are more broadly institutionalized and celebrated, particularly within universities, they will always be limited in their impact and accessibility.

To make these efforts sustainable, universities and funding agencies must recognize civic engagement as a core competency and invest in long-term capacity-building. Strengthening training pipelines, supporting grassroots networks, and integrating engagement into academic structures will cultivate a scientific workforce capable of advancing evidence, building trust, and contributing to a healthy democratic society. This is the future SNAP seeks to help build, and one that institutions should actively embrace and support.

## Supplementary Material

biag053_Supplemental_Files

## Data Availability

The data underlying this article are available in Zenodo at https://doi.org/10.5281/zenodo.19460337.
