# Supplementary material for: The Scientist Network for Advancing Policy (SNAP): building infrastructure for early-career scientists in policy and public engagement
Source: Bioscience. 2026 Jun 18;76(8):683–9. doi: 10.1093/biosci/biag053 (PMC13485995; doi:10.1093/biosci/biag053)
Supplement: Supplementary file 1 [file biag053_supplemental_files.zip › Supplemental Table 1.docx]

**Supplemental Table 1.** The modules and core objectives of SNAP’s modular, open-access Science Policy 101 curriculum.

| **Module** | **Objective** |
| --- | --- |
| Starting a Science Policy Group | Tips and tricks to start and sustain on-campus student science policy and science communication organizations with SNAP support. |
| Science Policy 101 | Introducing the structures of government and how science interfaces with policy. |
| How Government Works | Exploring the mechanisms of congressional committees, federal agencies, and the budgetary process. |
| Science Communication and Public Speaking | Learning how to frame scientific expertise for non-specialist audiences, from policymakers to community groups. |
| Science Diplomacy | Understanding how science informs foreign policy, enables international cooperation, and creates opportunities for scientists to engage with embassies and global institutions on shared challenges. |
| Writing for Policymakers | Learning about the different written documents that policymakers read, what they are used for, and how to write them. |
